# Supplementary material for: Complex multi-fault rupture and triggering during the 2023 earthquake doublet in southeastern Türkiye
Source: Nat Commun. 2023 Sep 9;14:5564. doi: 10.1038/s41467-023-41404-5 (PMC10492857; doi:10.1038/s41467-023-41404-5)
Supplement: Supplementary file 1 — Supplementary Information [file 41467_2023_41404_MOESM1_ESM.pdf]

Supplementary Materials for

Complex multi-fault rupture and triggering during the 2023 earthquake doublet in southeastern Türkiye

Chengli Liu[1*], Thorne Lay[2], Rongjiang Wang[1,3], Tuncay Taymaz[4], Zujun Xie[1], Xiong Xiong[1], Tahir Serkan Irmak[5], Metin Kahraman[6] & Ceyhun Erman[4]

[1]School of Geophysics and Geomatics, China University of Geosciences, Wuhan, Hubei, China.
[2]Department of Earth and Planetary Sciences, University of California Santa Cruz; Santa Cruz, CA, USA.
[3]GFZ German Research Centre for Geosciences, Potsdam, Germany.
[4]Department of Geophysical Engineering, The Faculty of Mines, İstanbul Technical University, Maslak 34467, Sarıyer, İstanbul, Türkiye.
[5]Department of Geophysical Engineering, Kocaeli University, Umuttepe 41380, Kocaeli, Türkiye.
[6]Eurasian Institute of Earth Sciences, İstanbul Technical University, Maslak 34467, Sarıyer, İstanbul, Türkiye.

*Corresponding author: C.L.: liuchengli@cug.edu.cn

This PDF file includes:

Supplementary Figs. 1 to 13.

Supplementary Tables 1 to 3.

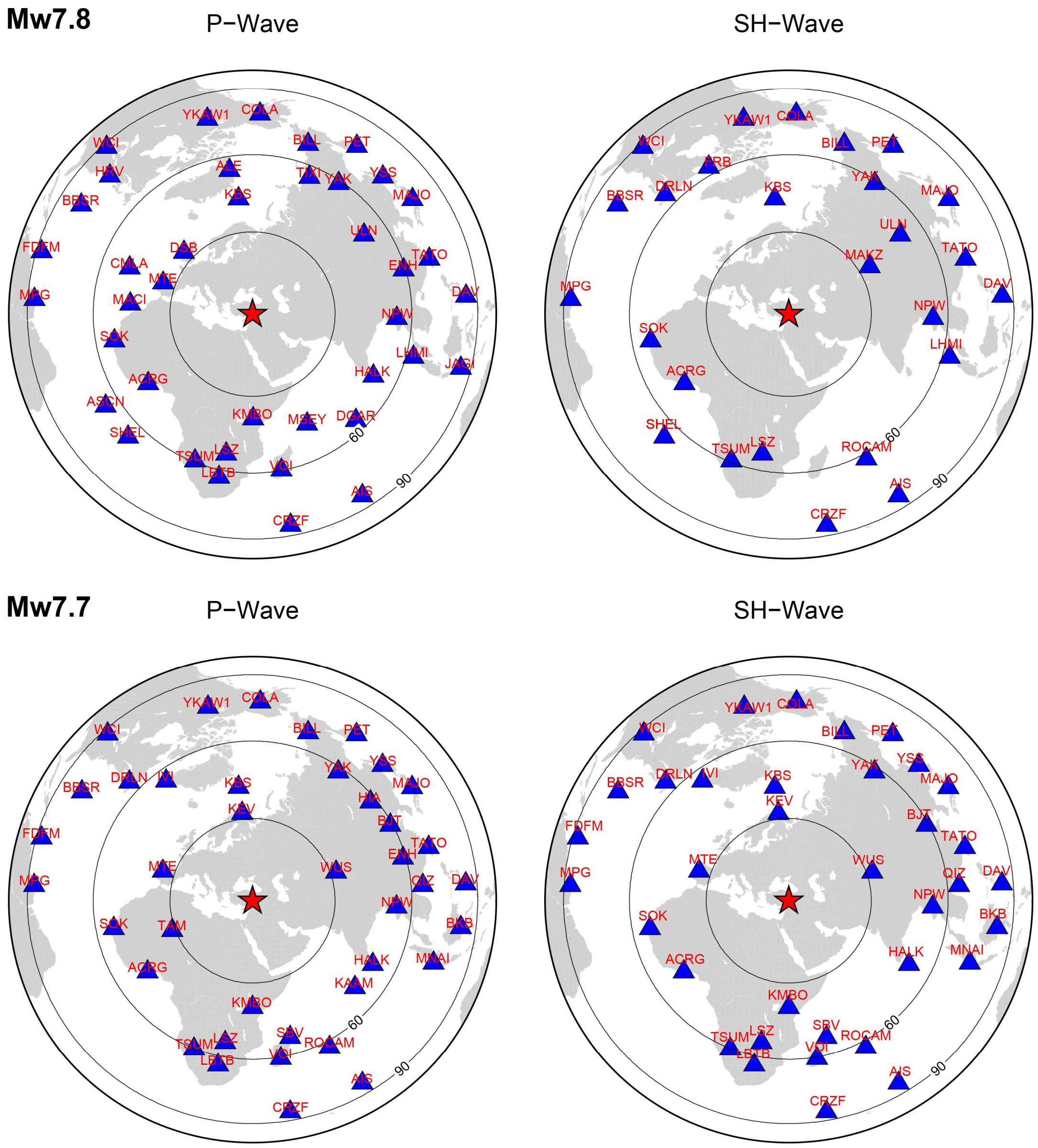

Supplementary Fig. 1. The teleseismic broadband seismic station distributions of the 2023 Türkiye earthquake doublet utilized in the separate finite-fault inversions for the two events.

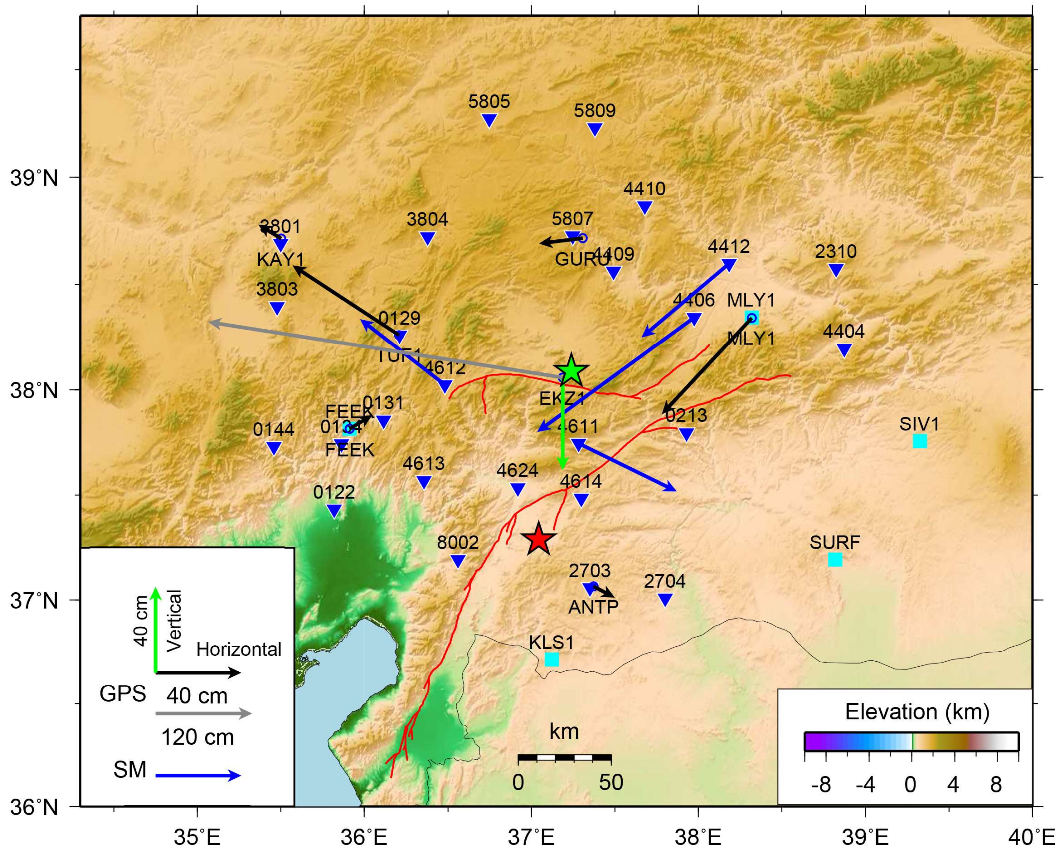

Supplementary Fig. 2. Distribution of strong-motion stations (inverted blue triangles) and GNSS stations (cyan squares and blue circles) for the M_W 7.7 event. Black and gray vectors indicate the horizontal GNSS static displacements, and the green vector indicates a vertical GNSS static displacement. Blue vectors show the horizontal coseismic displacements derived from strong-motion data. These data are used in the joint inversion. The epicenters of the M_W 7.8 and M_W 7.7 events are shown by the red and green stars, respectively, and the red lines represent positions of fault ruptures detected by post-earthquake satellite data.

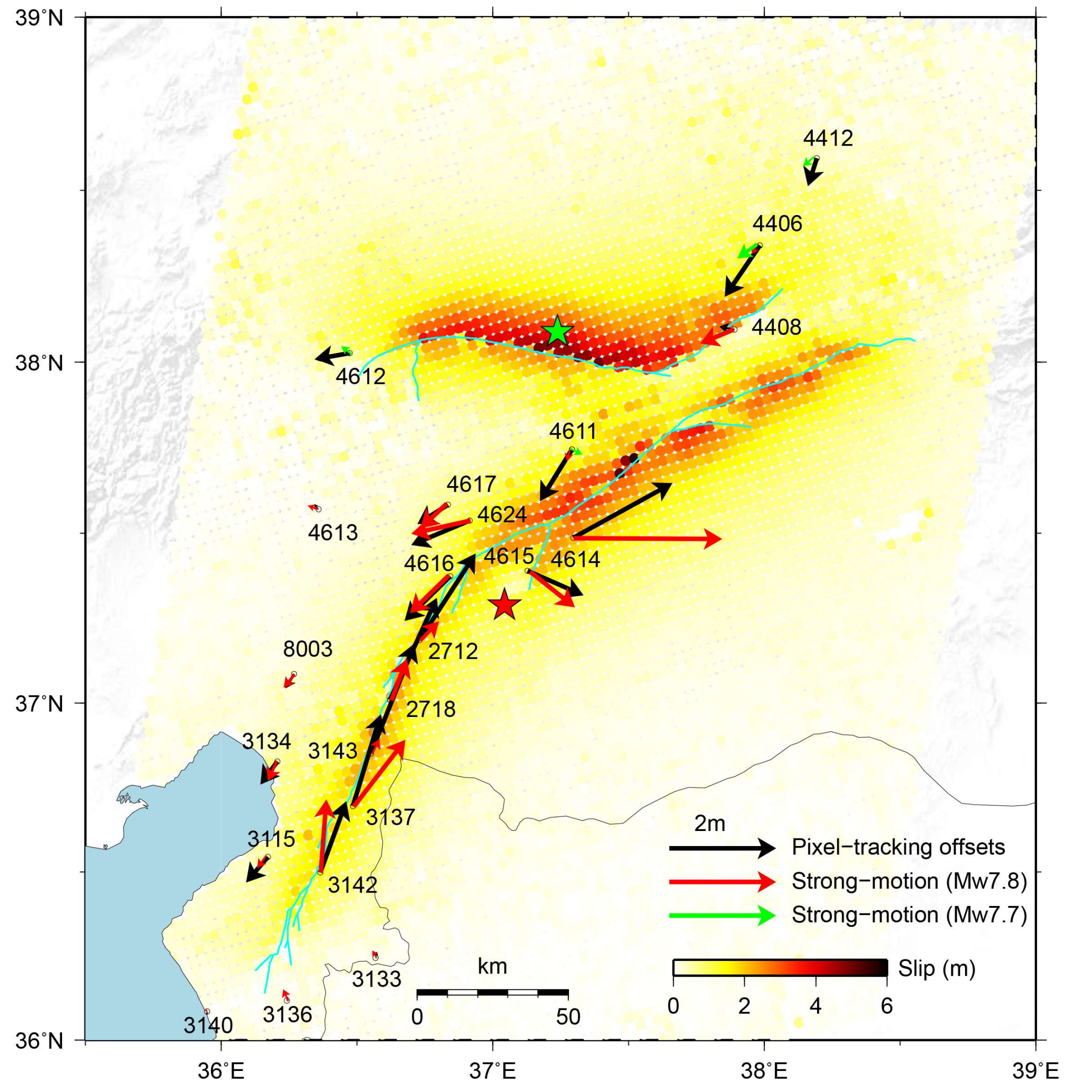

Supplementary Fig. 3. Comparisons between the coseismic displacements derived from the strong-motions of the earthquake doublet and the composite horizontal displacements derived from pixel-tracking offsets of Sentinel-1 satellite radar images. Cyan lines depict the positions of fault ruptures detected by post-earthquake satellite data. Black arrows represent the averaged horizontal displacements within a 1 km range, while the displacements derived from the strong-motion of the M_W 7.8 and M_W 7.7 events are indicated by red and green arrows, respectively. The red and green stars show epicenters of the M_W 7.8 and M_W 7.7 events, respectively.

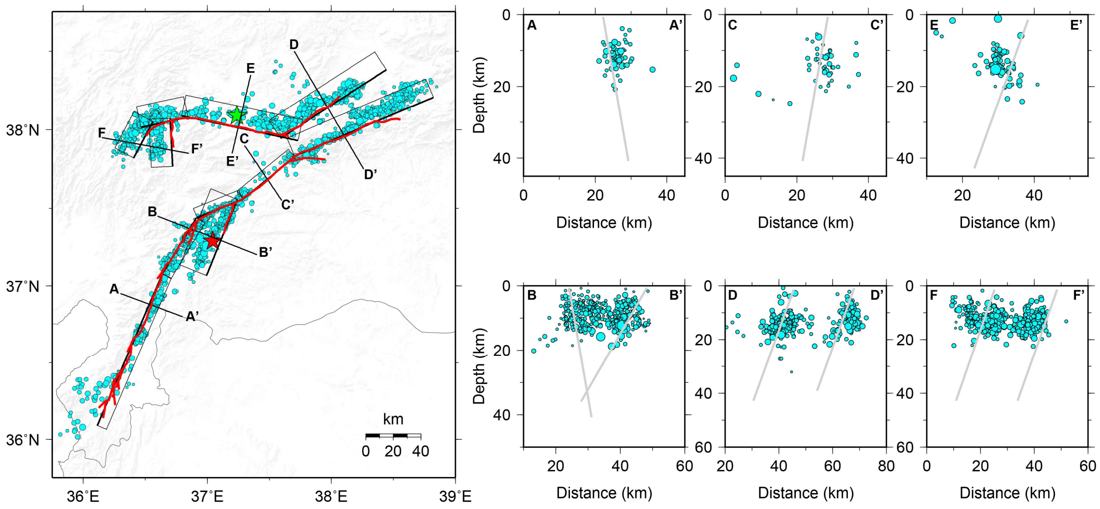

Supplementary Fig. 4. Map and cross-section display the distribution of relocated aftershocks along various profiles. Red and green stars represent the epicenters of the M_W 7.8 and M_W 7.7 events, respectively. Cyan-filled circles, sized proportionally to magnitude, indicate the relocated aftershocks with a magnitude greater than 1.0. The fault ruptures, identified through post-earthquake satellite data, are represented by red lines. Assumed fault segments are indicated by black rectangles, with the shallow (surface) edge depicted with thicker lines. In each cross-section, the gray lines denote the position and dip of the intersected fault segments.

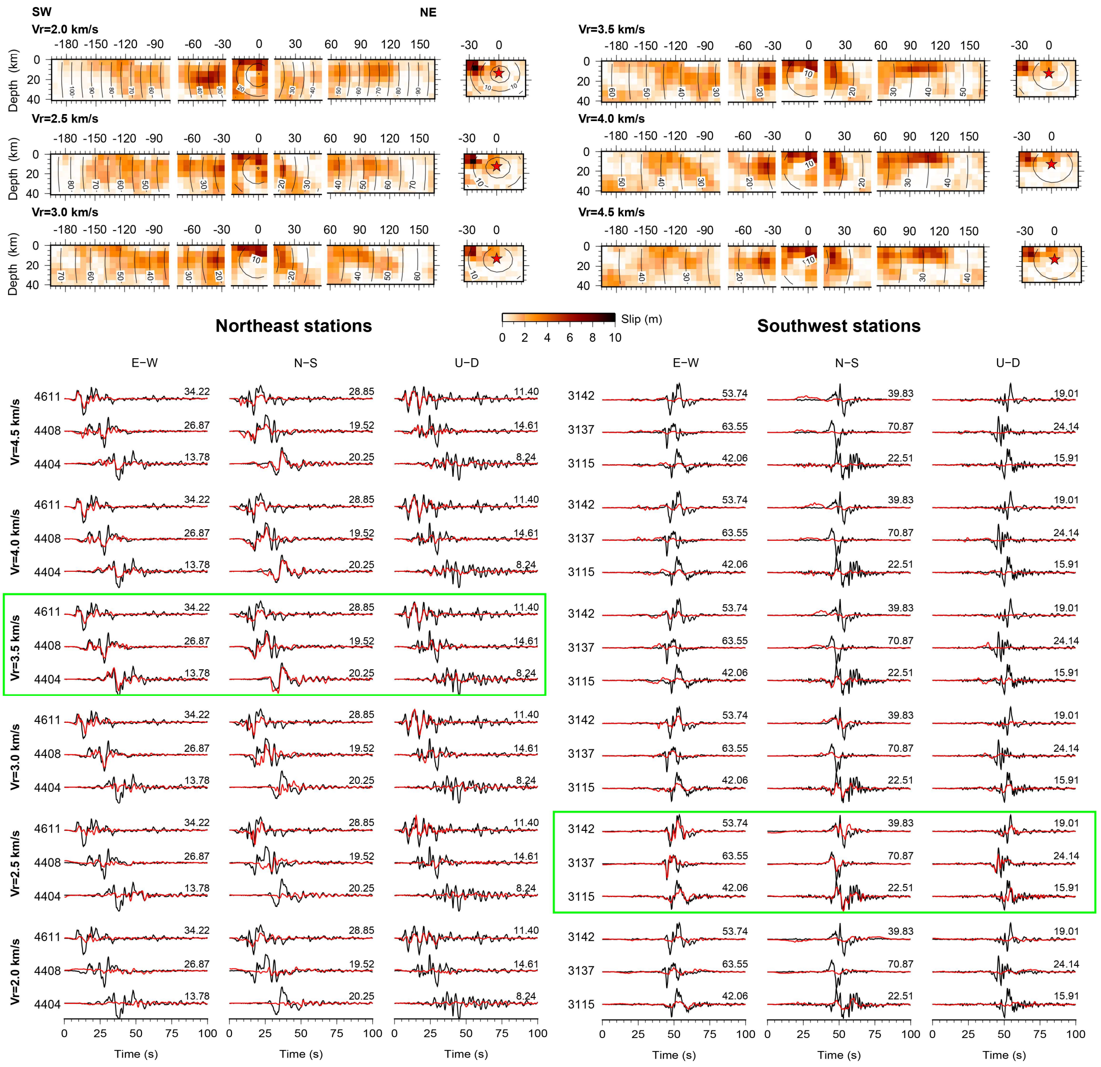

Supplementary Fig. 5. Tests of imposed uniform rupture velocity constraints. The upper panel displays the slip models obtained using specified rupture velocities ranging from 2.0 to 4.5 km/s over the entire model. The lower panels show the waveform fitting for the models with different rupture velocities. Representative stations to the northeast and southwest are displayed (see Fig. 2). The station names are shown to the left of each three-component record. Green rectangles outline waveform fits for optimal average rupture velocities, which vary in the northeast and southwest directions.

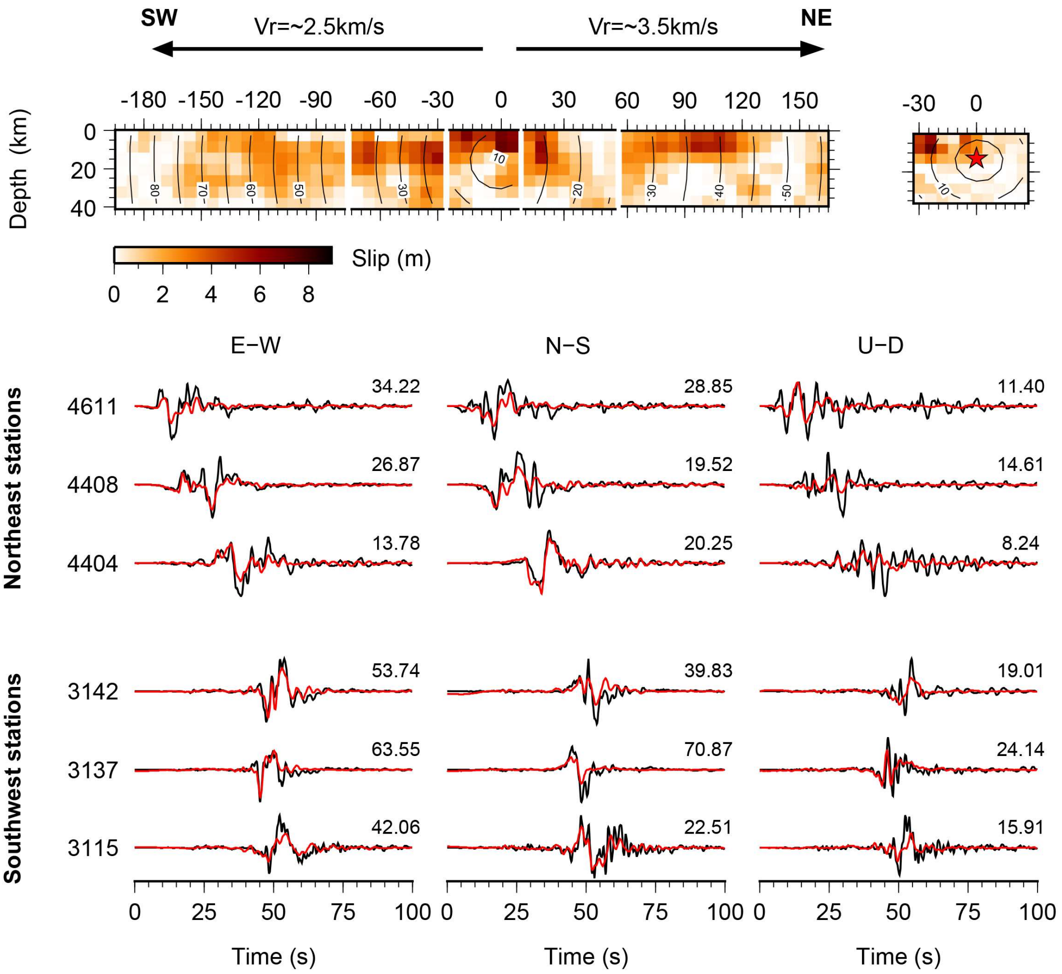

Supplementary Fig. 6. Rupture velocity test imposing different rupture velocities toward the northeast (3.5 km/s) and southwest (2.5 km/s) for one joint inversion for the M_W 7.8 event, resulting in good waveform fitting in both directions simultaneously.

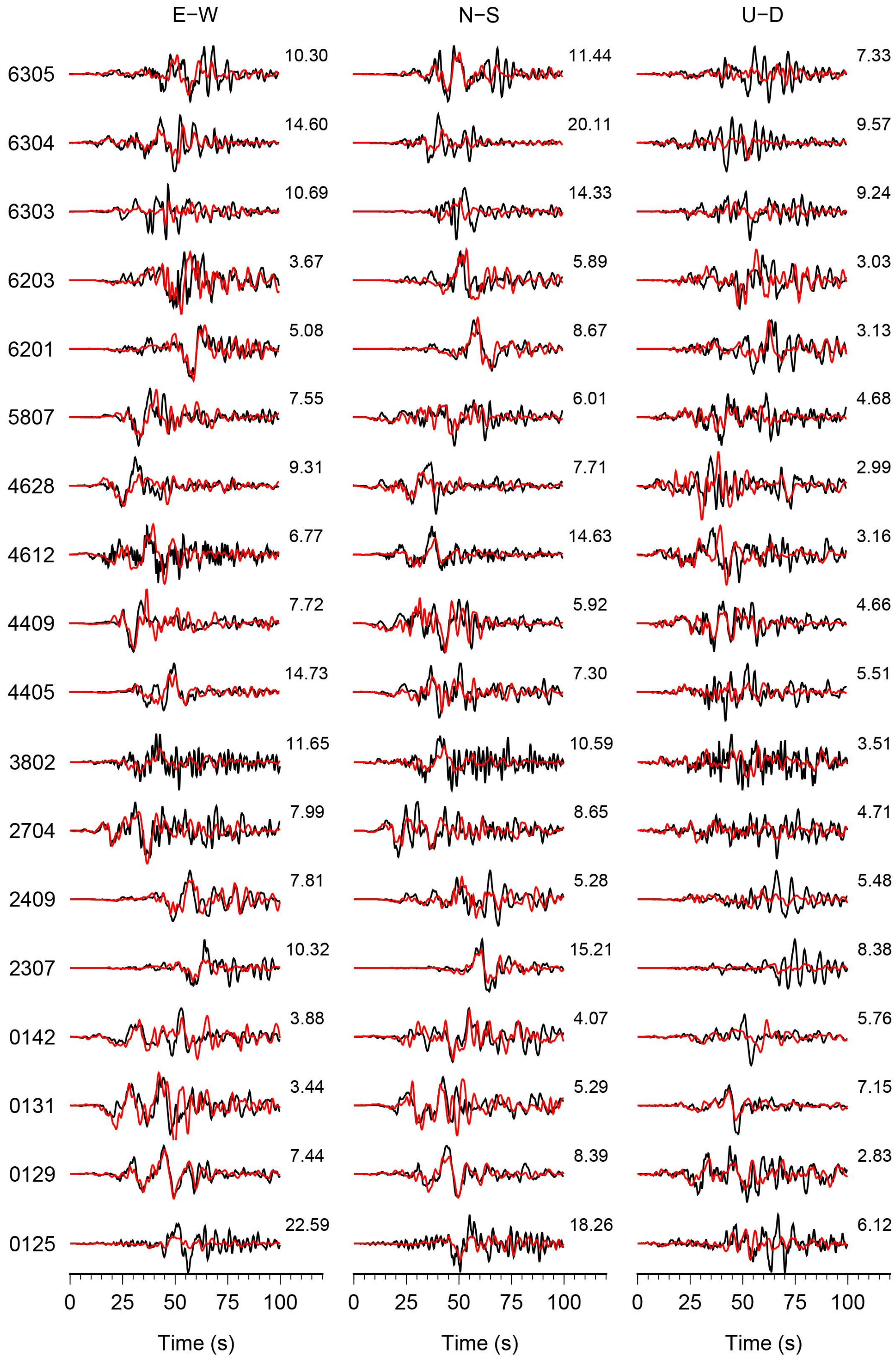

Supplementary Fig. 7. Comparisons of three-component strong-motion ground velocity observations (black) and synthetic seismograms (red) for the M_W 7.8 slip model in Fig. 3. Data and synthetics are aligned on the first P arrivals. The station name is listed on the left of each row; the numbers at the upper right of each waveform comparison indicate the maximum observed ground velocity in cm/s.

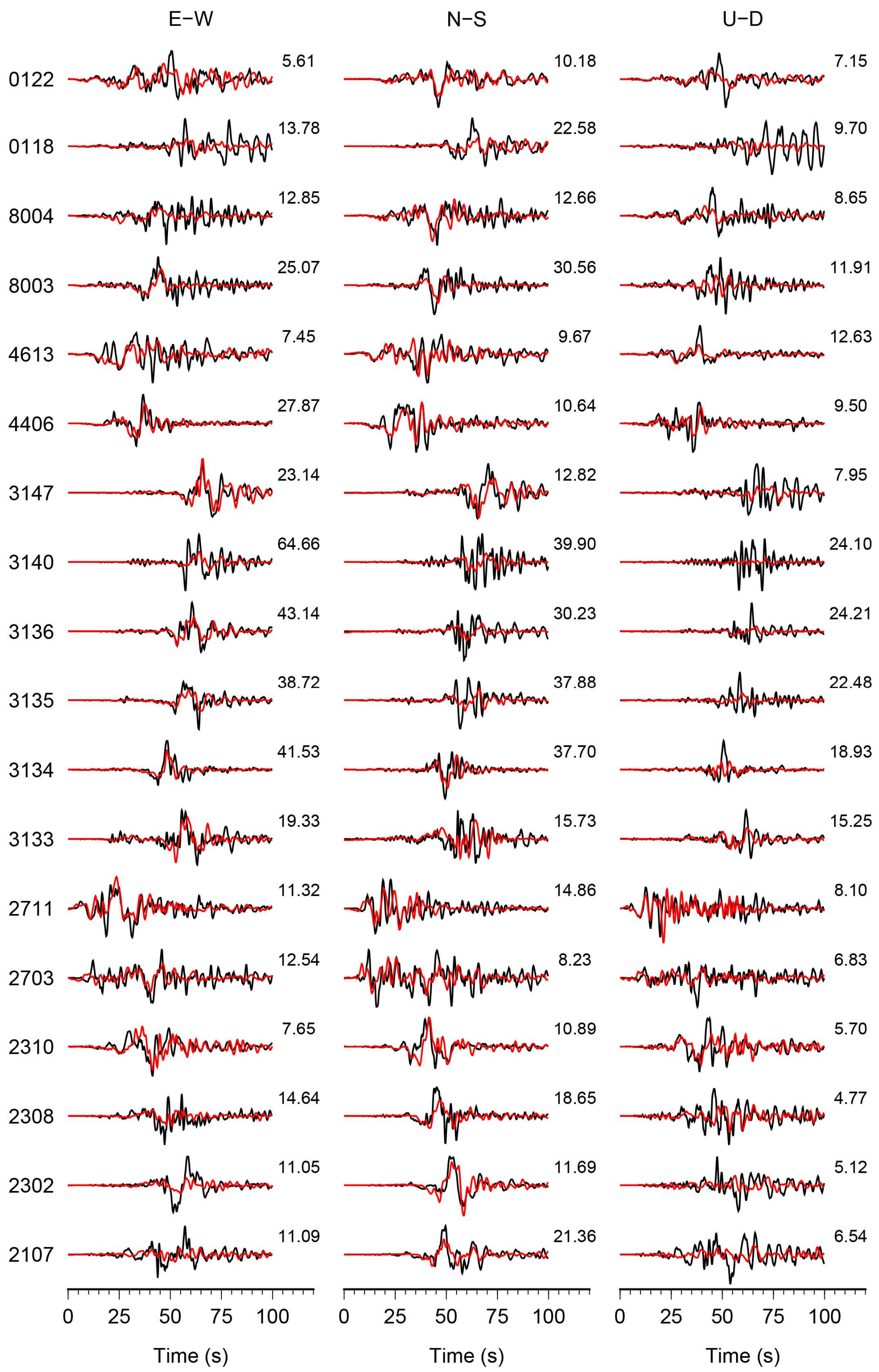

Supplementary Fig. 7. Continued.

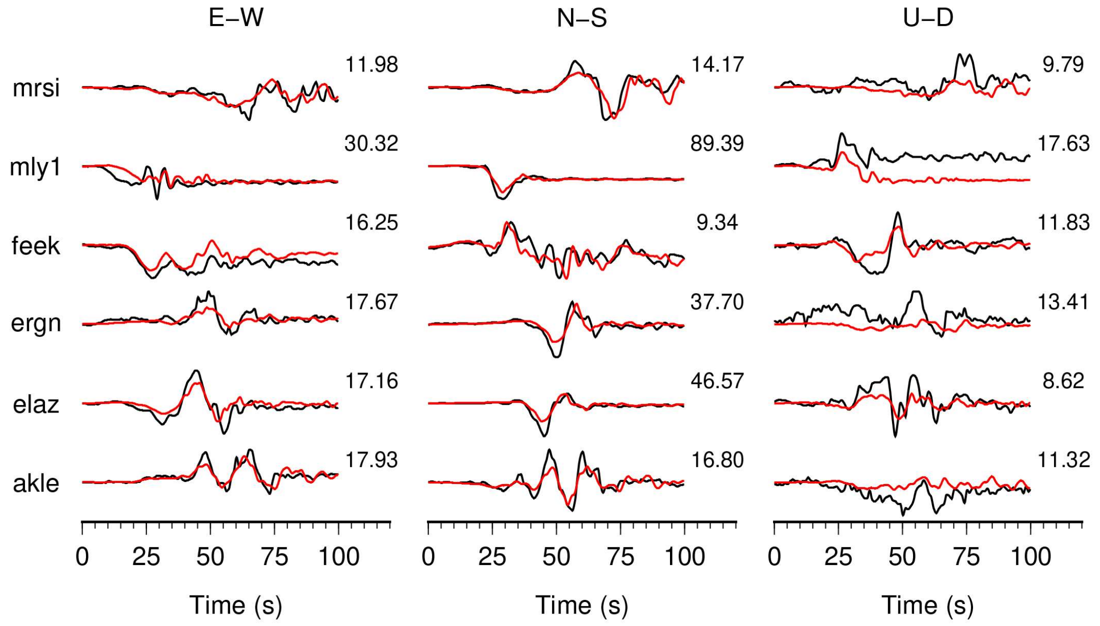

Supplementary Fig. 8. Comparisons of high-rate GNSS displacement time series (black) and synthetic seismograms (red) for the M_W 7.8 slip model in Fig. 3. Data and synthetics are aligned on the first P arrivals. The station name is listed on the left of each row; the numbers at the upper right of each waveform comparison indicate the peak observed displacements in cm.

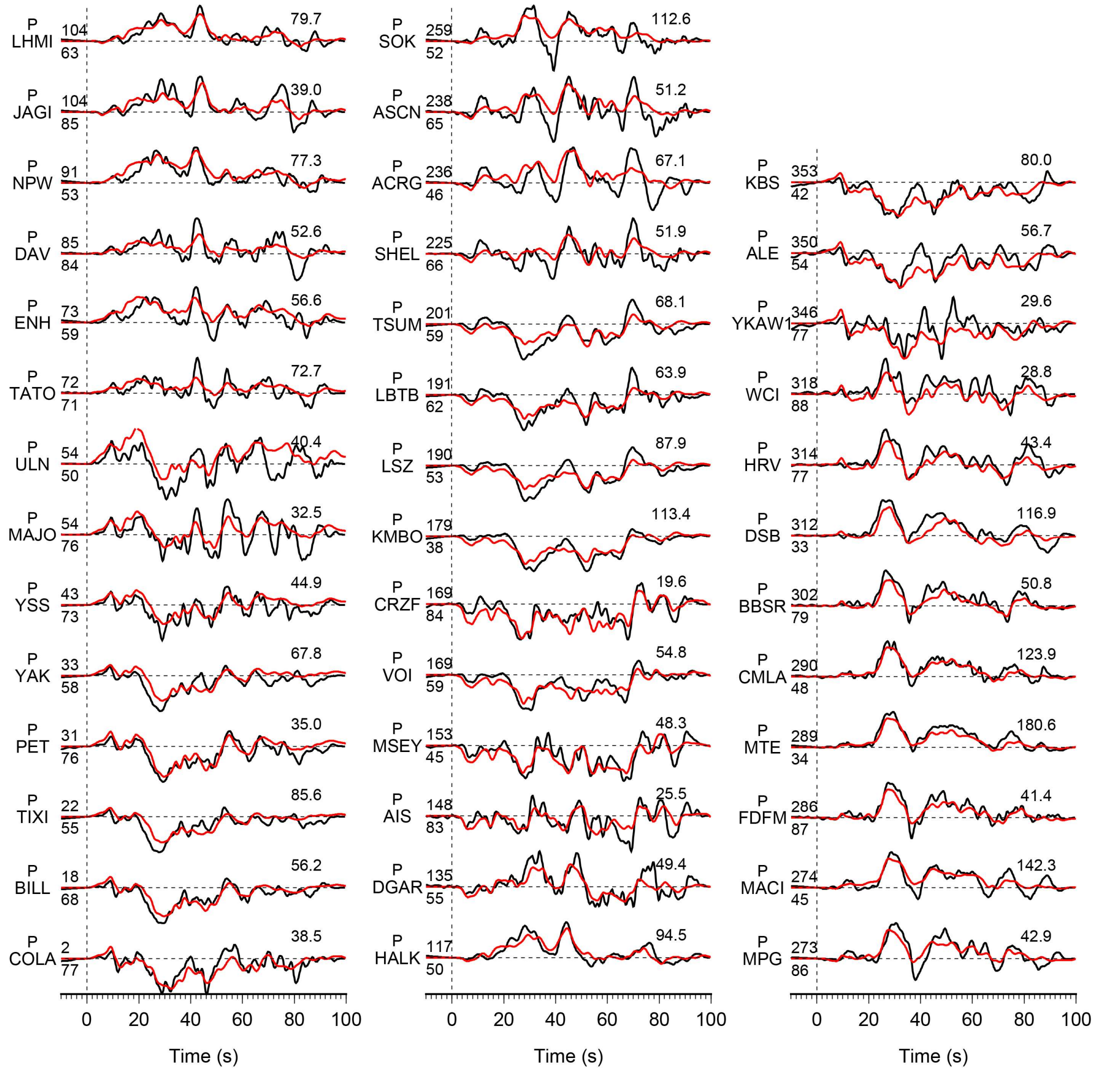

Supplementary Fig. 9. Comparison of observed (black) and synthetic (red) teleseismic P-wave ground displacements for the M_W 7.8 slip model in Fig. 3. Data and synthetic seismograms are manually aligned on the first arrivals. Station names and phase types are indicated on the left of each comparison. The azimuth (above) and epicentral distance (below) in degrees are shown at the beginning of each record. The number above the right portion of each comparison is the peak amplitude of the observed ground displacement in µm.

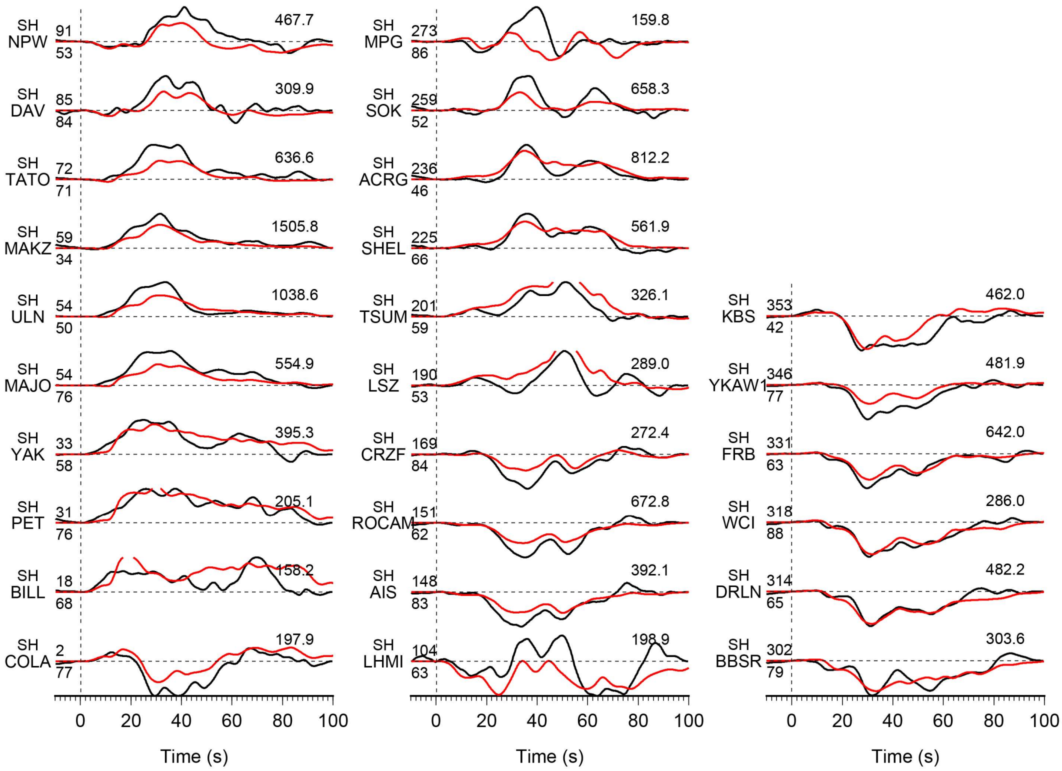

Supplementary Fig. 9. Continued, but for SH-waves.

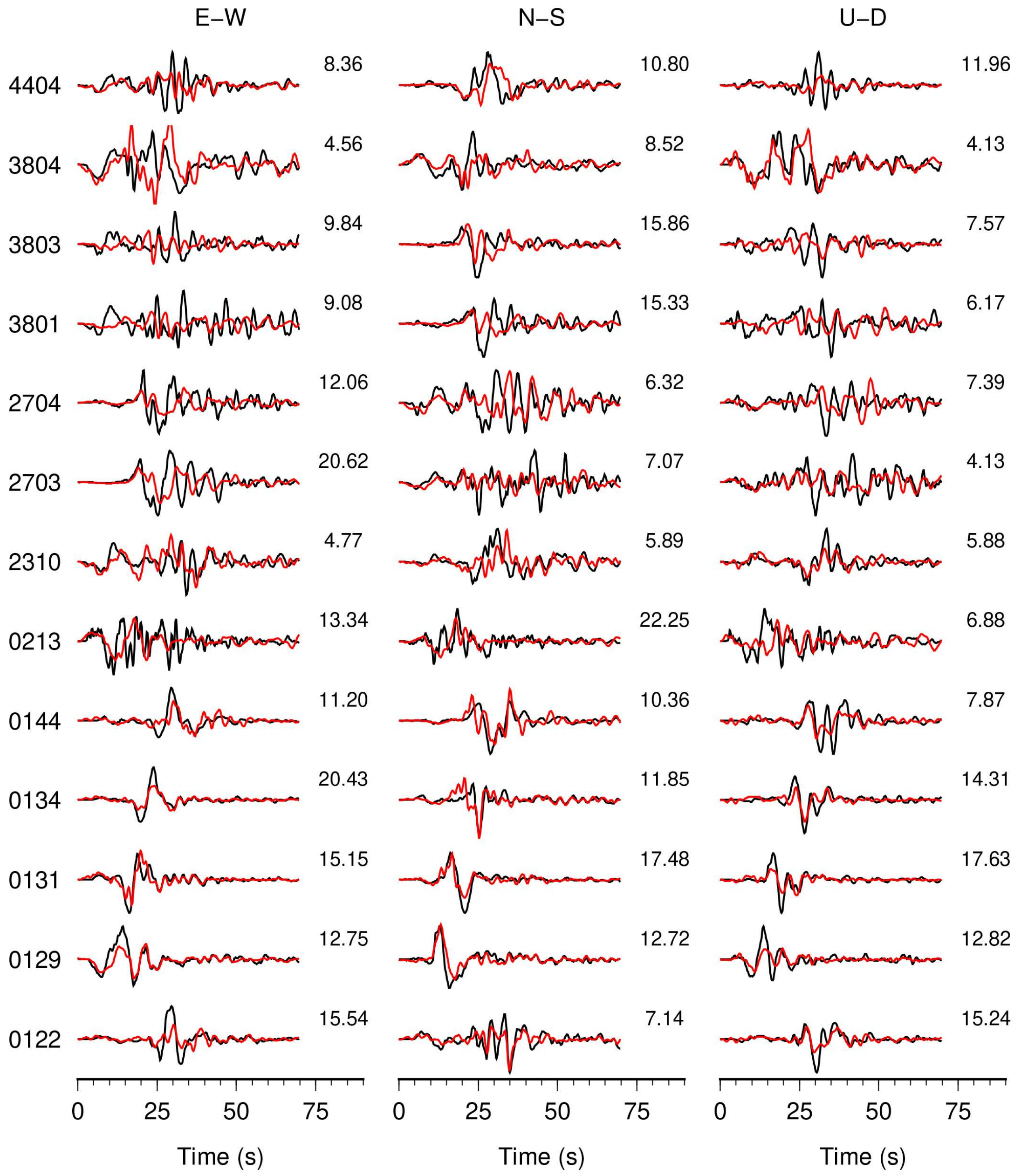

Supplementary Fig. 10. Comparisons of three-component strong-motion ground velocity observations (black) and synthetic seismograms (red) for the M_W 7.7 slip model in Fig. 5. Data and synthetics are aligned on the first P arrivals. The station name is listed on the left of each row; the numbers at the upper right of each waveform comparison indicate the maximum observed ground velocity in cm/s**.**

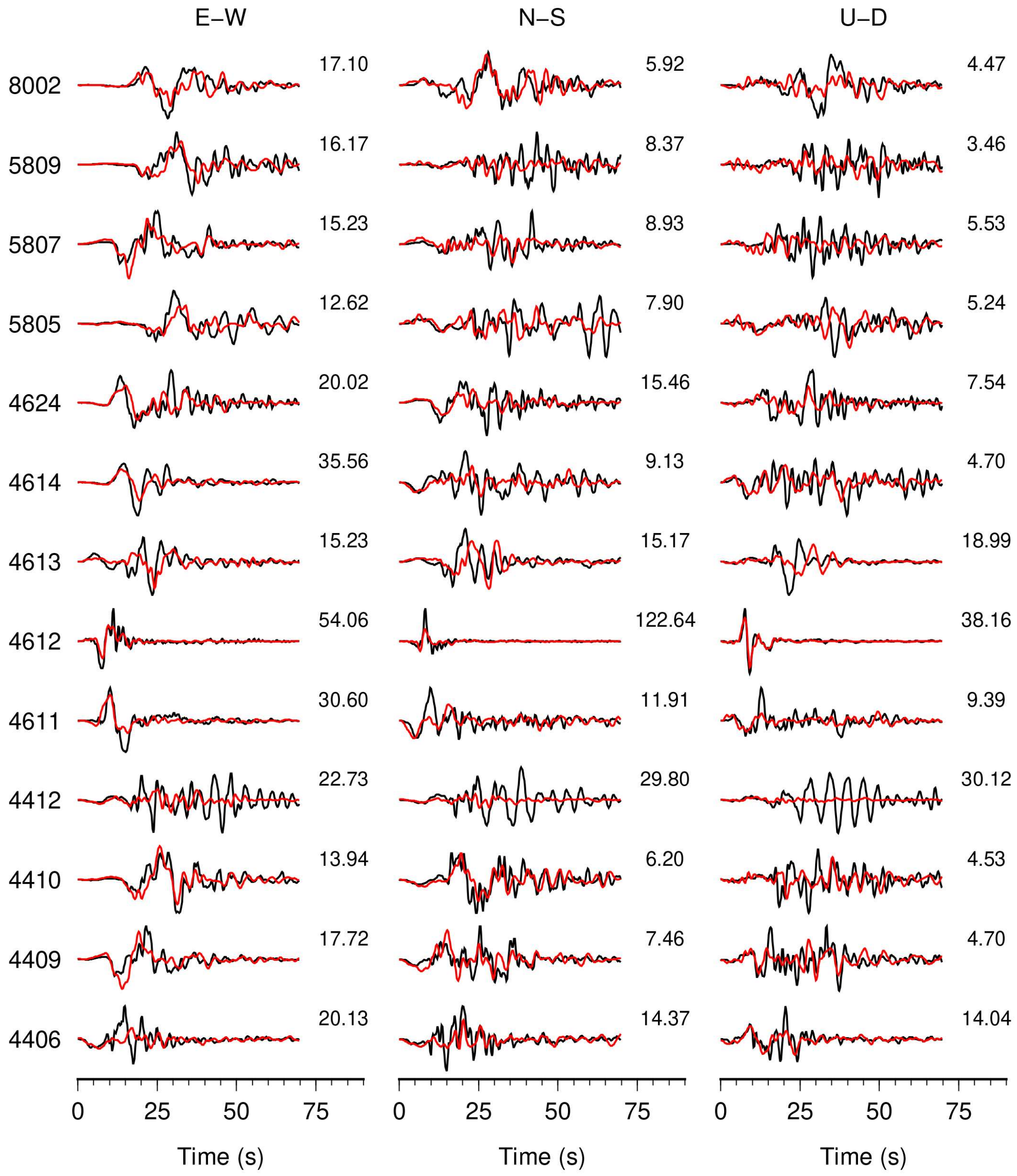

Supplementary Fig. 10. Continued.

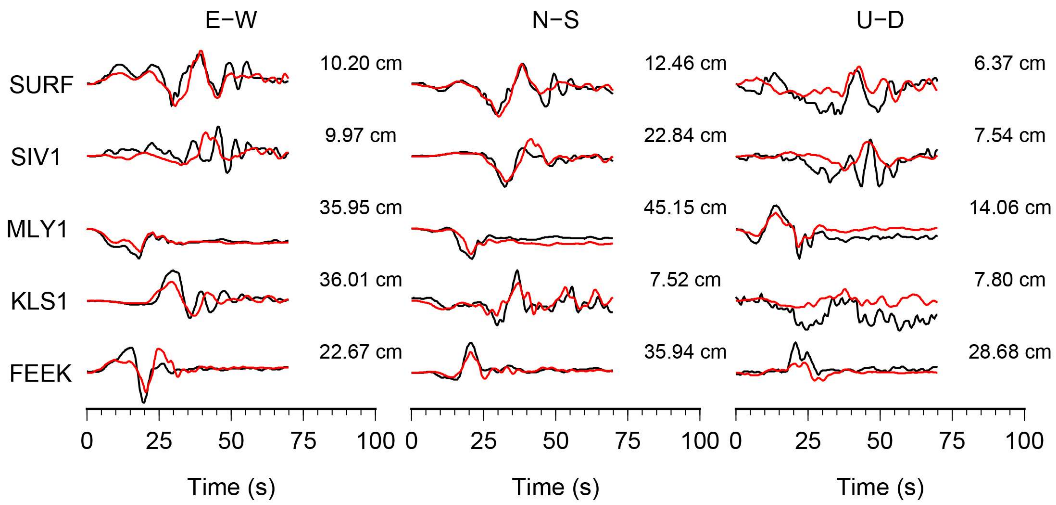

Supplementary Fig. 11. Comparisons of high-rate GNSS displacement time series (black) and synthetic seismograms (red) for the M_W 7.7 slip model in Fig. 5. Data and synthetics are aligned on the first P arrivals. The station name is listed on the left of each row; the numbers at the upper right of each waveform comparison indicate the peak observed displacements in cm.

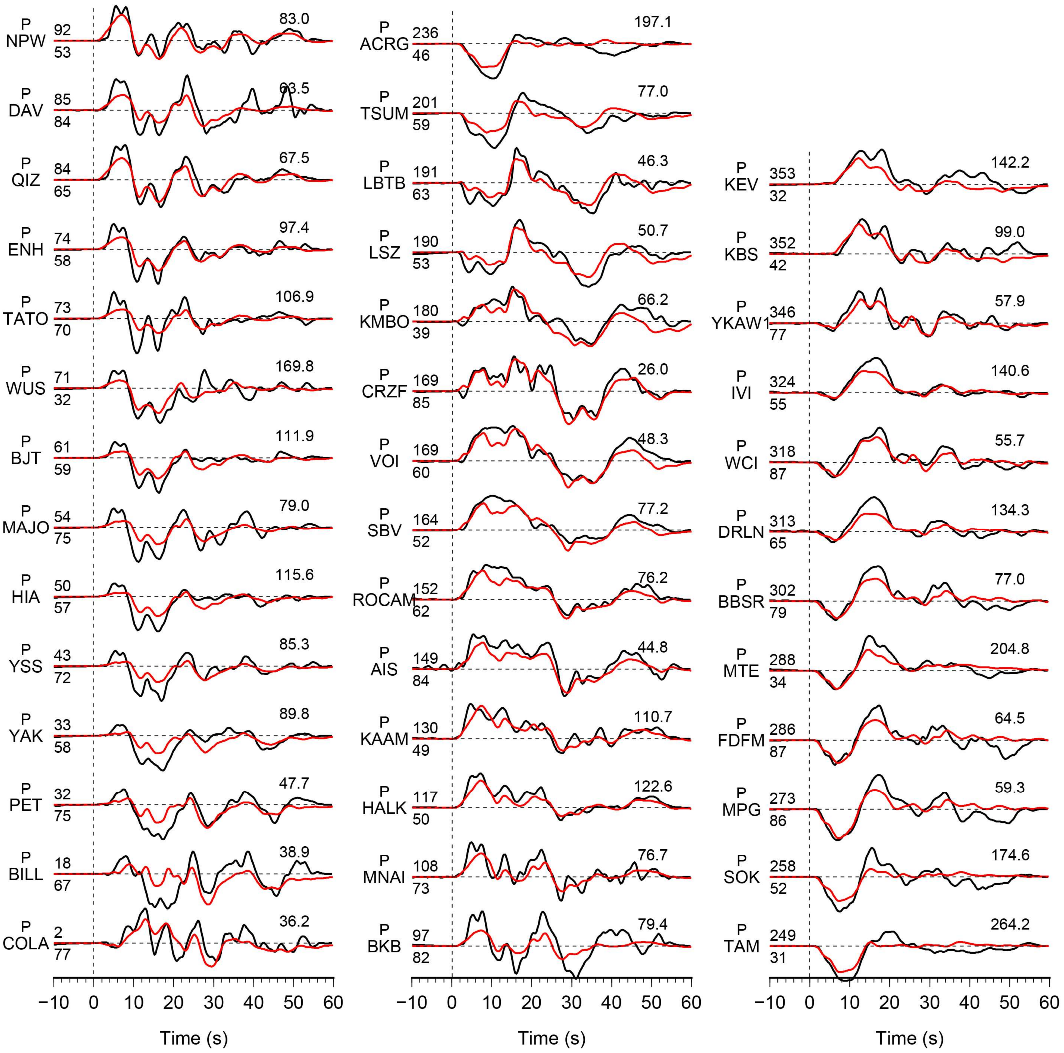

Supplementary Fig. 12. Comparison of observed (black) and synthetic (red) teleseismic P-wave ground displacements for the M_W 7.7 slip model in Fig. 5. Data and synthetic seismograms are manually aligned on the first arrivals. Station names and phase types are indicated on the left of each comparison. The azimuth (above) and epicentral distance (below) in degrees are shown at the beginning of each record. The number above the right portion of each comparison is the peak amplitude of the observed ground displacement in µm.

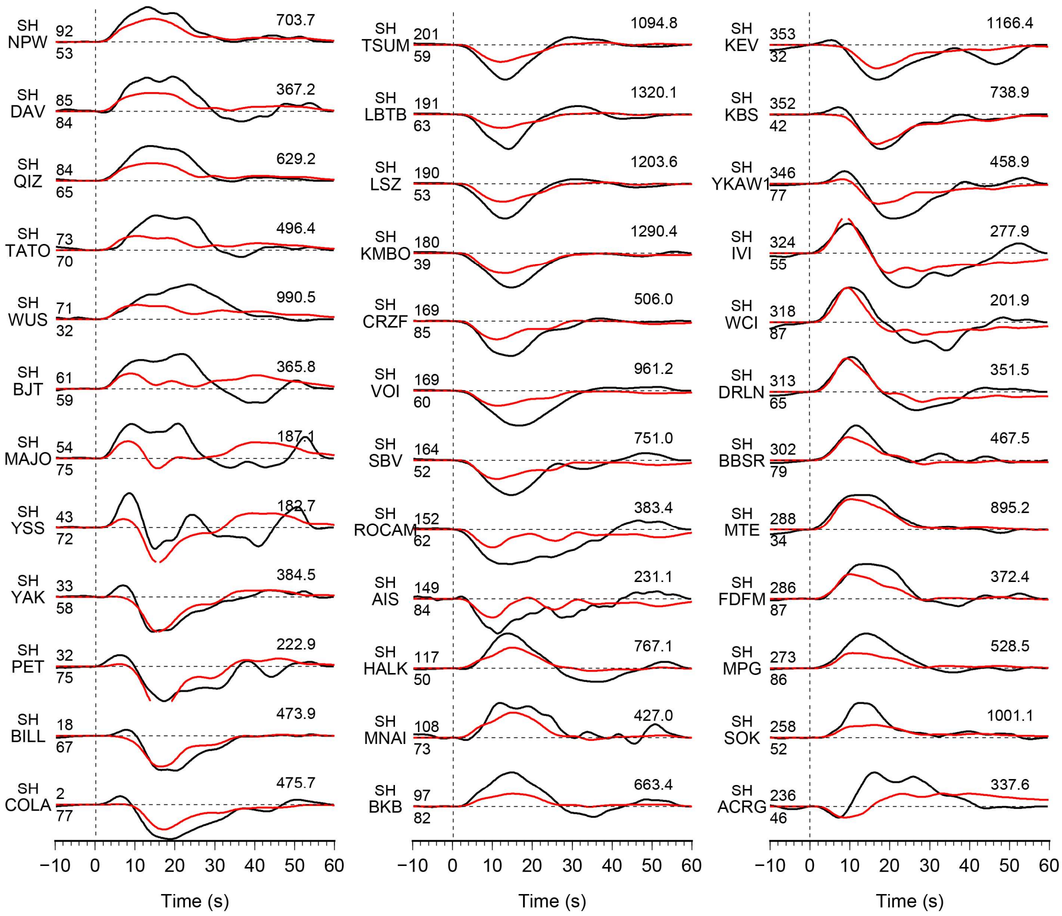

Supplementary Fig. 12. Continued, but for SH-waves.

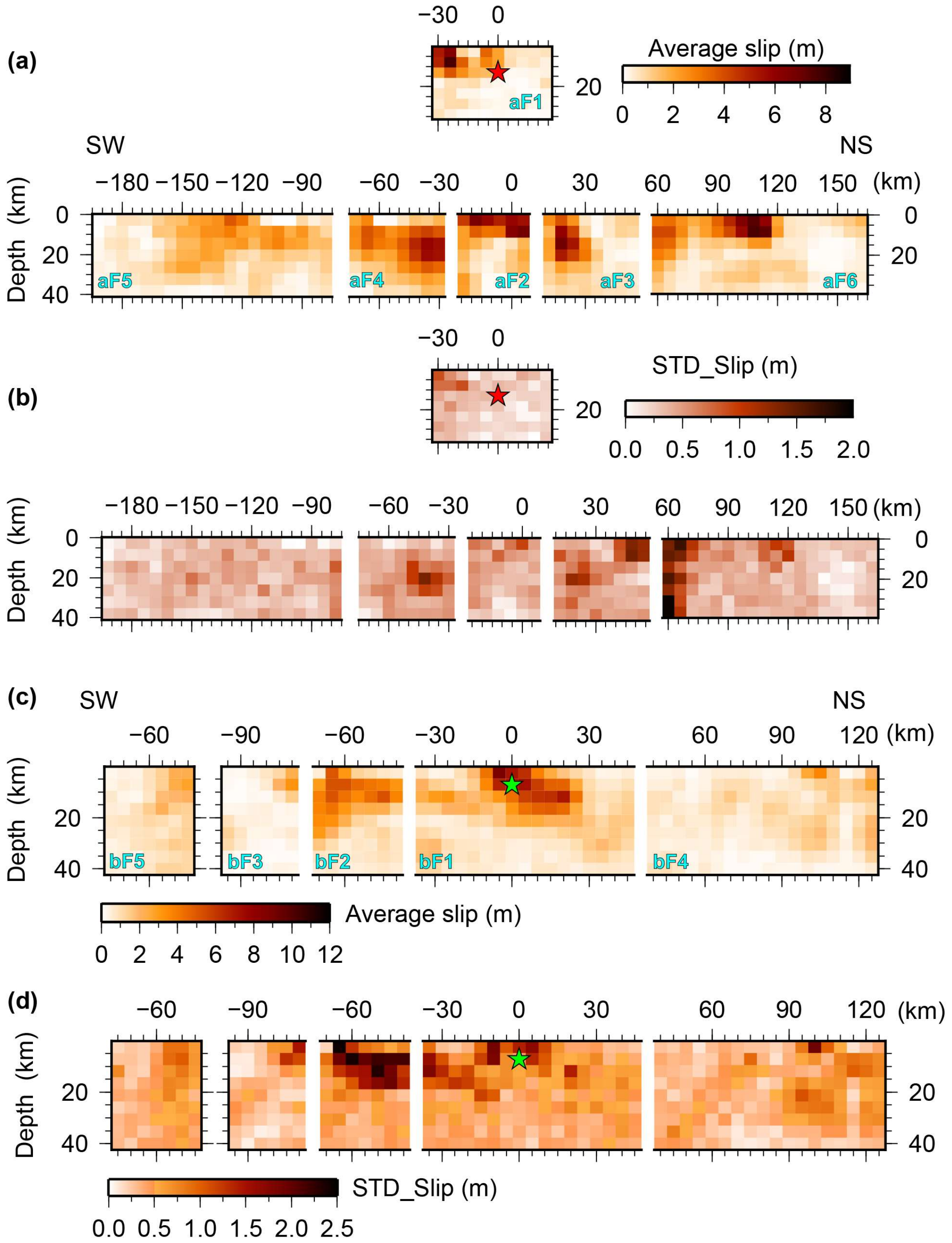

Supplementary Fig. 13. The average slip distribution (a) and (c), and standard deviation estimates (STD) (b) and (d) of ten models with different random seeds for the M_W 7.8 event and the M_W 7.7 event, respectively. The red and green stars show epicenters of the M_W 7.8 and M_W 7.7 events, respectively.

Supplementary Table 1. The coseismic displacements derived from the strong motion data.

The M_W 7.8 event					
SM Station	Latitude	Longitude	E-W (m)	N-S (m)	U-D (m)
4615	37.3868	37.1380	0.834	-0.665	-0.689
4616	37.3755	36.8384	-0.729	-0.710	-0.122
4624	37.5361	36.9176	-1.108	-0.246	
2712	37.184	36.7328	0.345	0.356	
4614	37.4851	37.2977	2.780	-0.014	
4617	37.5855	36.8303	-0.498	-0.465	
2718	37.0078	36.6266	0.293	0.776	
4611	37.7472	37.2843	-0.060	-0.242	
4613	37.5701	36.3574	-0.195	0.069	
3143	36.8489	36.5571	0.131	0.273	
8003	37.0842	36.2694	-0.205	-0.251	
3137	36.6929	36.4885	0.964	1.242	
3134	36.8276	36.2048	-0.168	-0.372	
3142	36.4980	36.3661	0.105	1.357	
3115	36.5463	36.1646	-0.165	-0.222	
4408	38.0962	37.8873	-0.627	-0.262	
3133	36.2432	36.5736	-0.083	0.145	
4406	38.3439	37.9738	-0.098	-0.192	
3136	36.1159	36.2472	-0.094	0.221	
3140	36.0816	35.9498	-0.044	0.071	
The M_W 7.7 event					
SM Station	Latitude	Longitude	E-W (m)	N-S (m)	U-D (m)
4611	37.7472	37.2843	0.229	-0.112	
4612	38.0239	36.4819	-0.198	0.154	
4406	38.3439	37.9738	-0.367	-0.268	
4412	38.5969	38.1838	-0.205	-0.173	

Supplementary Table 2. Fault geometry parameters of the 2023 Türkiye earthquake doublet used in the joint inversion.

	Fault-segment parameters of the M_W 7.8 event					
	F1	F2	F3	F4	F5	F6
Strike	202°	248°	231°	31°	23°	247°
Dip	60°	80°	80°	80°	80°	70°
	Fault-segment parameters of the M_W 7.7 event					
	F1	F2	F3	F4	F5	
Strike	282°	258°	208°	237°	177°	
Dip	70°	70°	70°	70°	70°	

Supplementary Table 3. Ranges of the source parameters allowed for each subfault during the joint inversion.

	The M_W 7.8 event	The M_W 7.7 event
Slip (m)	(0.0, 20.0)	(0.0, 20.0)
Rake (°)	(-45, 45)	(-90, 90)
Rise time (s)	(2.4, 24)	(1.6, 16)
Velocity (km/s)	(1.5, 4.5)	(2.0, 5.0)
